# Remarks upon the Medical Properties and Therapeutical Application of Veratrum Viride

**Published:** 1857-03

**Authors:** V. H. Taliaferro

**Affiliations:** Atlanta, Georgia


### ARTICLE IV.

*Remarks upon the Medical Properties and Therapeutical Applications of Veratrum Viride.* By V. H. TALIAFERRO, M. D., Atlanta, Georgia.

American Hellebore, or Veratrum Viride, anterior to the investigations of Dr. Osgood, was supposed to be analogous, in its therapeutic properties, to that of the European variety.

Dr. Osgood discovered that its effects upon the system were materially different from the white hellebore of the dispensatory, in not producing the drastic effects upon the stomach and bowels, or the alarming prostration so characteristic of the latter variety.

But, instead of this, found it to be wholly destitute of cathartic properties, having no tendency to produce irritation of the lining membranes of the intestinal canal, and reducing the heart's action to as low as 35 beats per minute. In consequence of this difference, Dr. Osgood considered it as possessing very superior advantages over the European hellebore. He does not, however, seem to have fully appreciated its peculiar virtues. The profession were, at least, not inclined to test the remedy to any great extent, at his suggestion. This was owing, no doubt, to the prejudices entertained to the ordinary veratria, the previous history of which, at once condemned its use.

Since that time, the properties of Veratrum Viride, or American Hellebore, have, by the minute investigations of Dr. Norwood, of S. C., been more fully brought to light. We believe that he has added an imperishable boon to the profession in thus bringing before them, a remedy of such inestimable properties.

Its power alone, over the vascular system, will ultimately assume, for its deserved position, upon the pages of pharmacy. We say deserved, because we consider it as classing among those great therapeutic agents which must live through the lapse of time.

We propose, first, to notice its most important properties, and the diseases to which it is most applicable. Dr. Norwood gives it not less than eight distinct properties—its most important, being vascular, sedative, nauseant, emetic, diaphoretic, and nervine.

The first observable effect, after its use, is upon the circulation; reducing the number and force of the pulsations of the heart, thereby, under all circumstances, lessening febrile excitement. If its use now be continued, nausea follows, and finally, vomiting; which is free and easy, accompanied with muscular relaxation, and very great reduction of the heart's action.

The emesis, which follows its use, is invariably unattended with spasmodic action, which so frequently accompanies the use of ordinary emetics. As a nauseant and emetic, then, we see at once, the class of diseases, to which it is best suited; as in croup, bronchitis, laryngetis, asthma, pleurisy, pneumonitis, &c. From the high vascular and nervous excitement, which characterizes this class of diseases, the most prompt and energetic antiphlogistic treatment is requisite. Foremost among these, the lancet has ever been considered the great indispensable—that its substitute is found in Veratrum, we shall endeavor to prove.

It is objected to by many, on account of the dangerous and alarming prostration succeeding its use. This seems to be the great cry raised against it. So powerful an agent, too—so poisonous in over-doses—should never be left in the hands of nurses, thereby hazarding the lives of our fellow-creatures. Upon the very same ground, must we not lay aside those old and established remedies, which, for centuries past, have proven to be of such inestimable value in the amelioration of disease? Must we cease the use of such remedies as arsenic, mercury, opium, antimony, &c., because their incautious administrations produces alarming symptoms? The same course of reasoning would preclude the idea of ever giving a remedy, at all, that would, in over-doses, produce death, unless directly from the physician's hands.

These objections arise mainly, not from experiment or philosophical reasoning, but a natural prejudice existing in the profession, to the use of new remedies.

In regard to its being so prostrating a remedy, we have only to say, that we have never seen any prostrating effect resulting from its use, unless injudiciously administered in large and inordinate doses, which we consider no more lessens its value than the effect of coma, resulting from opium, depreciates its value; or that arsenic is less serviceable because it produces death.

That these remedies are by far more hazardous in large doses than Veratrum, we cannot for a moment doubt, because our experience and observation teaches us that Veratrum will not, in any single dose, produce death. It is no doubt owing to the fact, that it is so readily expelled from the system; the stomach being unable to retain it sufficiently long to endanger life.

We do not pretend to say that it would not produce death by continued repetition ot large doses, thereby exhausting the patient with continued emesis.

* Dr. Newsome attended two patients, who had each taken a drachm, with the intention of producing abortion, followed by no alarming symptoms, save very great nausea and vomiting. This is an immense dose of a medicine, requiring only from 6 to 10 drops to produce its effect.

A physician of this city swallowed an ounce through mistake, at one draught, attended only with extreme nausea and vomiting, and some difficulty in breathing.

It seems to have no tendency in inordinate quantites, to inflame the stomach as most emetics.

Its most remarkable virtue is its influence over the heart's action, making it a most appropriate remedy in the treatment of a variety of diseases; and also, one of the most prompt and efficient antiphlogistics in use.

It is superior to antimony as an antiphlogistic, from the fact, that it is wholy free from drastic properties.

---

* Dr. Newsome, of Reynolds, Georgia, Nashville Journal, January, 1857.

It is superior to ipecacuanha and other emetics, because of its influence over the circulation which is obtained without nausea.

It is superior to the use of the lancet, because its effects are more decided, and the "vital fluid" is retained, carrying on its important office of conveying nutrient material to the various tissues, which is an important consideration in protracted cases of disease.

We contend that it is not only unnecessary, but injudicious to abstract blood, whether or not inflammatory action exists, when the same effect may be produced by the administration of remedial agents. That this is effected in the use of Veratrum Viride, no one who has ever fully and fairly tested its virtues, will deny.

The great advantage it possesses over the abstraction of blood, is that its effects are far more decided, and not followed by the reaction which almost invariably succeeds venesection. Not only this, but the effects of the remedy may be continued for days, and even weeks, successively, without unpleasant sequences, whereas, a frequent repetition of blood-letting would, in most cases, be injurious.

Physicians, generally, are not disposed to lay aside remedies to which, by long continued use, they have become attached; remedies with which they have for years battled with disease, to experiment upon the properties of new remedies.

While we condemn the idea of experimenting with the hordes of quack remedies which infest the land, we do believe that it is the duty of the profession, when a remedy is brought before them by respectable and scientific physicians, claiming for it important medical properties, such as is claimed for Veratrum, that its therapeutic properties should be fully and satisfactorily tested by the profession before it be condemned.

A remedy coming to us with the recommendation of such men as Watson, Frost, Osgood, Norwood, &c., is sufficient to elicit from the profession, the deepest interest in its investigation.

No pharmaceutical preparation claims the same power over the circulatory system. Digitalis, it is true, has its power over the heart's action, but the uncertainty and inefficiency of that action is obvious to every physician familiar with its effects.

Unlike Digitalis, the use of Veratrum is sure to be followed by premonitory symptoms, as nausea, &c., previous to a very great reduction of the circulation. Our experience with it, teaches us that it will not, under any circumstances, reduce the heart's action to less than thirty-five beats per minute.

To say then, that we have a remedy by which we can so successfully control the heart's action, as to confine it to any number of beats desired, is assuming for it, virtues unprecedented in the history of medicines. That it possesses such power over the vascular system, will not be doubted by those acquainted with its effects. If, in Veratrum, such virtues reside, we at once perceive the class of diseases to which it is peculiarly applicable, viz: Inflammatory affections. We would not, however, confine its field of action alone to this class of diseases; for it is an important adjuvant in the treatment of all affections connected with excessive vascular action. With Veratrum, we may confidently lay aside the lancet, and successfully treat the most active inflammations, which we consider preferable, in many respects, to the abstraction of large quantities of blood.

It is certainly of no little importance in all cases of disease, especially those which are protracted, where, from the small amount of nourishment taken into the system, the blood becomes deprived of its source of nutrient material, from which alone the various emunctories can be supplied, that we quiet, if possible, the excited state of the circulation without reduction of its quantity.

It is the only agent in the whole materia medica, by which we are enabled, without serious consequences to our patient,to quell the tempestuous beatings of the heart; the only remedy by which we can successfully check the constant supply of fuel to inflammatory action.

If these are its virtues, who will, for a moment, doubt its efficacy in the treatment of diseases, connected with abnormal action of the circulation, whether that action be primary or secondary.

In the treatment of pneumonia, it is unquestionably the most reliable agent we have. When the tissue of the lung becomes inflamed, febrile excitement running high, the breathing short and quick, the proper use of veratrum, soon quiets the excited action of the heart, and the blood is sent smoothly and gently along its channels. When this is accomplished, we cannot fear that rapid increase of inflammation in the lung tissue, so much dreaded in pneumonitis. The free use of Veratrum in the outset of the disease, will very frequently, at once, cut short its progress. Yet, while we place our principal reliance upon it, in the treatment of pneumonia, we believe that important adjuvants are found in the use of opium, aconite and calomel.

In this connection, we will make allusion to a case very recently treated by us with Veratrum.

A gentleman of this city, having been severely attacked with a chill, followed by high fever, requested that we should immediately see him. We found him some few hours after his chill, with very high febrile excitement; skin burning hot; pain in the limbs; severe headache, with injected conjunctiva. The pulse was beating over one hundred; very full and bounding—a fine pulse, indeed, for the use of the lancet—disposition to cough almost incessantly, attended with some little expectoration, and severe pain in the right side; auscultation revealed inflammatory action in the lower portion of the lobe of the right lung. We at once administered to him 10 drops of Veratrum, to be followed in three hours, by six, and in three hours more, if not nauseated, by five drops more; after which, we saw him in the following condition: pulse beating about 70; skin pleasant and moist; pain not so intense; free from nausea. We now gave him six grains of calomel, combined with ten grains of pul. doveri., that he might rest well through the night.

The Veratrum was continued through the night, in four drop doses. The ensuing morning, we found him with no fever; pulse beating naturally; and expectoration free and easy; free from pain except when coughing; auscultation revealed an evident subsidence in the inflammatory action of the lung. It is unnecessary to trace farther the change from day to day; suffice it to say, that the Veratrum was continued with no other medicine, save a dover powder at night, with a rapid subsidence of all diseased action, so much so, that upon the fourth day of his attack, he was able to walk upon the street; his medicine, in the meantime, being continued. This patient was never the least nauseated.

Although we believe that Dr. Norwood makes Veratrum, in some degree, a "hobby," we cannot think him absurd in saying that "Veratrum is as much a specific in pneumonitis as quinine in intermittent fever."

Veratrum, it is true, requires, frequently, the assistance of other remedial agents before its effects upon the system are permanent; and thus it is with mercury, antimony, quinine, &c.

We will now casually notice its effects in the treatment of typhoid fever—a disease in which most physicians condemn its use ; even those who use it freely in inflammatory diseases.

The pathology of typhoid fever is, that it is non-inflammatory or asthenic in its character, while inflammatory action, in the course of the disease, may supervene. Very great muscular and nervous debility are expected soon to succeed its onset; and it is from this fact, that Veratrum is condemned, supposing it to produce the very thing so important to be avoided. This supposed effect of Veratrum arises from a mistaken idea of its therapeutic properties. It is free from any prostrating effect in ordinary doses; but, like other emetics, produces more or less prostration when vomiting occurs. We believe its sequelæ to the system, to be more pleasant than is usually attendant upon the use of emetics. An important diagnostic symptom of typhoid fever is its peculiar pulse, small, feeble and frequent, running frequently to as high as 140 beats per minute. We consider, then, that there is not a more important indication to be fulfilled in its treatment, than to control the high vascular excitement attending the disease. If this be done, we certainly lessen, very much, the probability of an unfavorable issue. Veratrum is not only serviceable in the commencement of typhoid fever, but is an important agent in the treatment of its latter stages, reducing, frequently, the feeble-fluttering pulsation of the heart from 140 to 70 or 80, thereby changing an abnormal to a normal circulation. It is not reasonable that we should expect, from this change, very great prostration of our patient.

By holding the heart in abeyance, the great organ of the vascular system, we certainly hold an influence over diseased action, in which that organ becomes unduly excited, which no other remedy or combination of remedies gives us. We would not wish to leave the impression, as recommending Veratrum alone in the treatment of typhoid fever. We know that circumstances connected with the patient thus affected, frequently call for the use of other agents.

In the treatment of puerperal fever, we would expect more favorable results from Veratrum than any other remedy; for, how can inflammation advance with the circulation kept far below the natural standard, surface pale and cool, and the blood flowing smoothly and tranquilly along all its channels?

Is Veratrum abortive? This question seems of late, to have produced some controversy in the profession, in which we think it has been very satisfactorily proven to possess no power over the uterine organs. We would rather expect from its relaxing effect upon the muscular system, in large doses, to prevent or retard uterine contractions. From our opinion of the therapeutic properties of Veratrum, we have never hesitated to use it in cases connected with pregnancy. This we have frequently done; and save one case, have never known its use followed by premature labor. That was in case of a lady seven months advanced in pregnancy, who suffered severe and protracted illness from typhoid fever. She used Veratrum for the first week of her attack, after which it was discontinued. In the latter stages of her disease, she was delivered of a living seven months fœtus, and recovered after protracted confinement. In this case, fully two weeks had elapsed, after Veratrum was taken, before delivery.

Emetics given to the extent of violent nausea and vomiting, from the spasmodic action of the abdominal muscles, may produce abortion, having at the same time no direct effect upon the uterus. Veratrum, under predisposing circumstances to abortion, administered to the extent of violent emesis, might, from mechanical action of the abdominal muscles, excite the uterus to action. While we have never witnessed such a result, we are inclined to consider such an one not improbable. In the treatment of diseases connected with pregnancy, we would use Veratrum with the same caution we would other emetics in carrying it to the effect of emises.

At some future time, we propose to continue these remarks with report of cases.

